# Effect of Platelet-Rich Plasma (PRP) on Dermal Skin Flap Survival: An Experimental Study in Rats

**DOI:** 10.7759/cureus.108243

**Published:** 2026-05-04

**Authors:** Efthymios D Basagiannis, Christos Damaskos, Nikolaos Garmpis, Alexandros Papalampros, Stylianos Kykalos, Nikolaos Nikiteas

**Affiliations:** 1 Department of Plastic and Reconstructive Surgery, Aleris Specialistkliniken, Stockholm, SWE; 2 Department of Renal Transplantation, Laiko General Hospital, Medical School, National and Kapodistrian University of Athens, Athens, GRC; 3 N.S. Christeas Laboratory of Experimental Surgery and Surgical Research, Medical School, National and Kapodistrian University of Athens, Athens, GRC; 4 Second Department of Propedeutic Surgery, Laiko General Hospital, Medical School, National and Kapodistrian University of Athens, Athens, GRC; 5 First Department of Surgery, Laiko General Hospital, Medical School, National and Kapodistrian University of Athens, Athens, GRC

**Keywords:** flap necrosis, flap survival, prp, random-pattern skin flap, rat model

## Abstract

Background: Distal necrosis remains a common limitation of random-pattern skin flaps due to progressive decline in perfusion and ischemia-reperfusion injury. Platelet-rich plasma (PRP) is a growth factor-enriched biologic that has shown promise in flap models, although preparation and composition vary. This study evaluated the effects of heterologous leukocyte-rich PRP (L-PRP) on random-pattern dorsal skin flap viability in rats.

Materials and methods: Sixteen female Wistar rats underwent elevation of a cranially based random-pattern dorsal skin flap (8 × 2 cm) with placement of a cellulose barrier to impede revascularization from the wound bed. Animals were assigned to two groups (n = 8 per group): control (flap elevation only) and local heterologous PRP. Flaps were monitored daily, and animals were euthanized on postoperative day 8. Distal flap tissue was harvested, fixed in 10% formalin, processed, and evaluated by H&E staining. Necrosis was quantified histologically. Normality was assessed using the Shapiro-Wilk. Between-group comparisons were performed using one-way ANOVA with Tukey post hoc testing. Necrosis severity categories were compared using chi-square testing.

Results: Necrosis values were consistent with normal distribution (Shapiro-Wilk p = 0.130). One-way ANOVA demonstrated a significant difference in necrosis among groups (F = 14.855, p < 0.001). Tukey post hoc testing identified significant differences between Group A and Group B (p < 0.001). Mean necrosis decreased across treatments (Group A: 0.82, Group B: 0.59). Group A showed only mediocre/severe necrosis with no mild cases, while Group B showed no severe necrosis; the necrosis category depended significantly on group (χ^2^ = 24.924, p < 0.001).

Conclusions: Local heterologous L-PRP improved distal flap viability under the tested conditions and produced the lowest necrosis values compared to the control group. The absence of overt adverse reactions supports the feasibility of heterologous L-PRP in this setting.

## Introduction

Random-pattern dermal skin flaps remain indispensable in reconstructive surgery, yet distal flap necrosis continues to be a common limitation because perfusion progressively declines toward the distal territory. Experimental flap models show that failure is driven by a combination of reduced microvascular inflow, venous congestion, endothelial dysfunction, inflammation, oxidative stress, and ischemia-reperfusion injury, which together impair wound-bed integration and tissue viability [1,2].

Platelet-rich plasma (PRP) is plasma with a platelet concentration higher than baseline, typically defined as approximately three- to five-fold above physiological levels (commonly around 1-1.5 million platelets/µL) to achieve a biologically active formulation [3]. Platelets, derived from megakaryocytes, contribute not only to hemostasis but also to immune regulation and tissue repair. Their relevance to regenerative strategies derives largely from alpha-granules, which store multiple growth factors and signalling molecules, including transforming growth factor beta (TGF-β), insulin-like growth factor (IGF), epidermal growth factor (EGF), vascular endothelial growth factor (VEGF), connective tissue growth factor (CTGF), and others, as well as chemokines and adhesion-related mediators. Upon activation and degranulation, these factors can influence endothelial cells, fibroblasts, and mesenchymal populations, promoting cell migration, proliferation, and extracellular matrix production in the injured environment [3,4].

A major limitation in PRP research is the heterogeneity of preparation methods and product composition. The commonly used double-spin approach separates blood components in an initial low-speed centrifugation followed by a higher-speed centrifugation step to concentrate platelets. Standardization efforts, including frameworks such as DEPA (Dose of injected platelets, Efficiency of production, Purity of the PRP, Activation of the PRP) classification, highlight the need to report dose and composition parameters and help interpret outcome variability across studies and devices [5]. Comparative work indicates that double-spin protocols generally yield higher platelet concentrations than single-spin methods [6]. Protocol performance is influenced by centrifugation parameters, with recommended forces and durations proposed to maximize platelet yield while maintaining viability [7]. Additional technical variables may affect platelet activation state and product quality, including temperature (platelets can be activated at room temperature under certain conditions and excessive thermal manipulation can impair bioactivity) [8,9], choice of anticoagulant (with acid citrate dextrose solution A (ACD-A) commonly favoured over alternatives due to platelet viability considerations) [10], blood collection technique and needle or catheter dimensions (smaller diameters can promote premature activation) [11], the interval between blood draw and centrifugation [12], centrifuge configuration (horizontal versus vertical rotor performance), and decisions around exogenous activation. Activation method affects the release of bioactive molecules [13], and in some dermatologic indications, exogenous activation may not be beneficial [14,15]. These considerations are particularly relevant when PRP is applied in flap research, where the therapeutic target is microvascular perfusion and wound-bed integration.

Experimental data support PRP as a potential flap-salvage adjunct, though results vary by model and protocol. PRP has been reported to increase survival of skin flaps in rats at later time points (for example, postoperative days 5 and 7) compared with control [16]. In dogs, locally injected PRP improved perfusion and flap survival and reduced swelling, though effects on angiogenesis and collagenesis were not always statistically significant, and collagen-related findings have differed across studies [17]. In cats, PRP produced modest improvements with clearer effects on oedema than on survival [18]. Rabbit models have demonstrated improved viability alongside reduced inflammatory infiltration and increased vessel density [19], and free-flap or complex flap settings have shown increased VEGF and TGF-beta consistent with enhanced neoangiogenesis and flap adherence [20]. In a stringent rat model using an ultra-long dorsal random flap (8 × 2 cm), PRP improved survival, reduced inflammatory cell populations, increased vascular density and angiogenic growth factors (VEGF, PDGF), and increased nitric oxide levels, providing a mechanistic bridge between perfusion support and inflammatory modulation [21].

Accordingly, the present study evaluated distal flap necrosis in a rat random-pattern dorsal skin flap model after local administration of heterologous PRP prepared under a standardized double-spin protocol, compared with flap elevation alone. Because the final PRP product was not directly characterized for platelet concentration, leukocyte composition, or growth factor content, the intervention should be interpreted as a protocol-defined preparation rather than a composition-defined biological dose. Therefore, the findings are intended to reflect the effects associated with the specific preparation and administration conditions used in this experiment.

## Materials and methods

All experiments were conducted at the N.S Christeas Laboratory of Experimental Surgery and Surgical Research, Medical School, National and Kapodistrian University of Athens, Greece. All procedures were performed in accordance with applicable animal welfare regulations and were approved by the local ethics committee (Veterinary Service of the Prefecture of Attica, Greece - approval no.: 1348419/16-12-2022). A total of 16 female Wistar rats (body weight 202-277 grams) were used. Following surgery, animals were housed in specifically arranged cages under stable conditions of temperature and light-dark cycling and received a standard pellet diet (dry cylinders) and water ad libitum. Animals were monitored daily throughout the study period. Platelet and leukocyte counts of the final PRP preparation were not performed. This was due to the limited available donor blood volume, the intention to minimize additional handling and atmospheric exposure of the PRP before administration, and the potential technical limitations of platelet counting in highly concentrated PRP preparations.

Study design

Animals were divided into two experimental groups (n = 8 per group, n = 16 in total): (i) Group A (control): flap elevation without further interventions, and (ii) Group B (heterologous PRP): flap elevation followed by subcutaneous injection of heterologous PRP into the flap.

Histological assessment

The primary histological outcome was the continuous necrosis percentage. Histological sections from the distal flap were examined under a light optical microscope for ischemic and necrotic changes. Histological assessment in the present study was focused on the extent of necrosis percentage in the distal flap sections. A formal semiquantitative scoring of additional qualitative features, such as neoangiogenesis, collagen organization, microthrombi, or inflammatory cell subtype composition, was not performed. Histological assessment was performed by an observer blinded to the experimental group allocation.

Anesthesia and perioperative preparation

**Figure 1:**
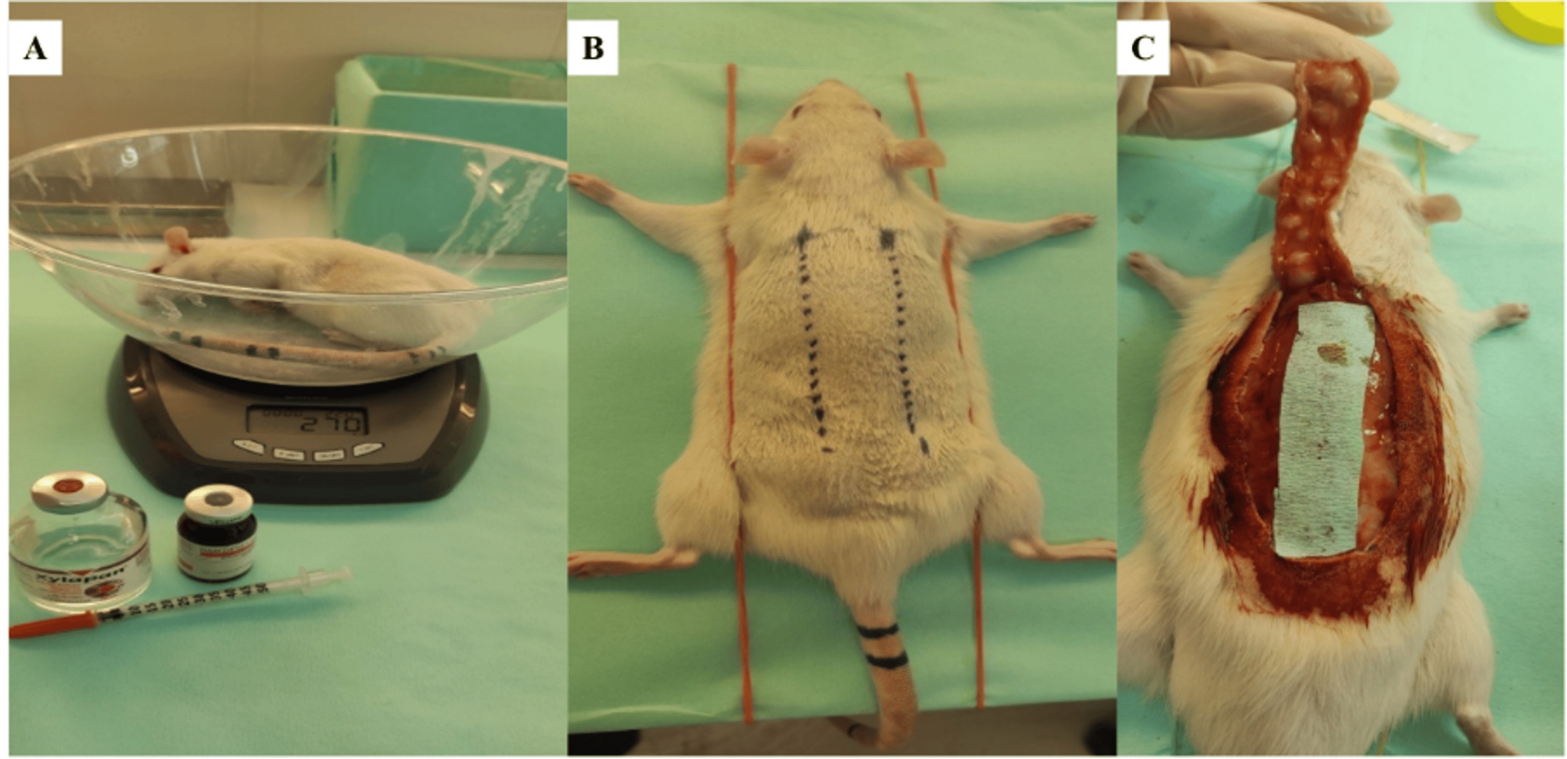
Experimental workflow for the random-pattern dorsal skin flap model. A: Animal weighing and anesthetic preparation; B: Preoperative marking of the cranially based 8 × 2 cm dorsal random-pattern flap; C: Flap elevation and placement of a cellulose barrier beneath the flap to prevent revascularization from the wound bed.

General anesthesia was induced by intraperitoneal administration of ketamine HCl (50 mg/kg) and xylazine HCl (10 mg/kg). Rats were weighed and transferred to the operating room (Figure 1A). The dorsum was shaved, prepared with povidone-iodine (Betadine), and draped in a sterile surgical field.

Random-pattern dorsal flap model and surgical procedure

A cranially based unipedicled random-pattern dorsal skin flap measuring 8 × 2 cm (length-to-width ratio 4:1) was designed on the back of each rat (Figure 1B). This ratio exceeds commonly recommended clinical random-pattern proportions (2:1-3:1) and was selected to evaluate distal necrosis under severe ischemic conditions. The flap was elevated using a scalpel and scissors in a plane superficial to the muscle fascia, without including fascia (Figure 1C). A cellulose sheet was placed under the flap to impede revascularization from the wound bed, ensuring that perfusion was derived primarily from the cranial pedicle. The flap was then returned to its original anatomic position and sutured with 3-0 Vicryl (Ethicon Inc., Somerville, NJ, USA).

Preparation of heterologous PRP

**Figure 2:**
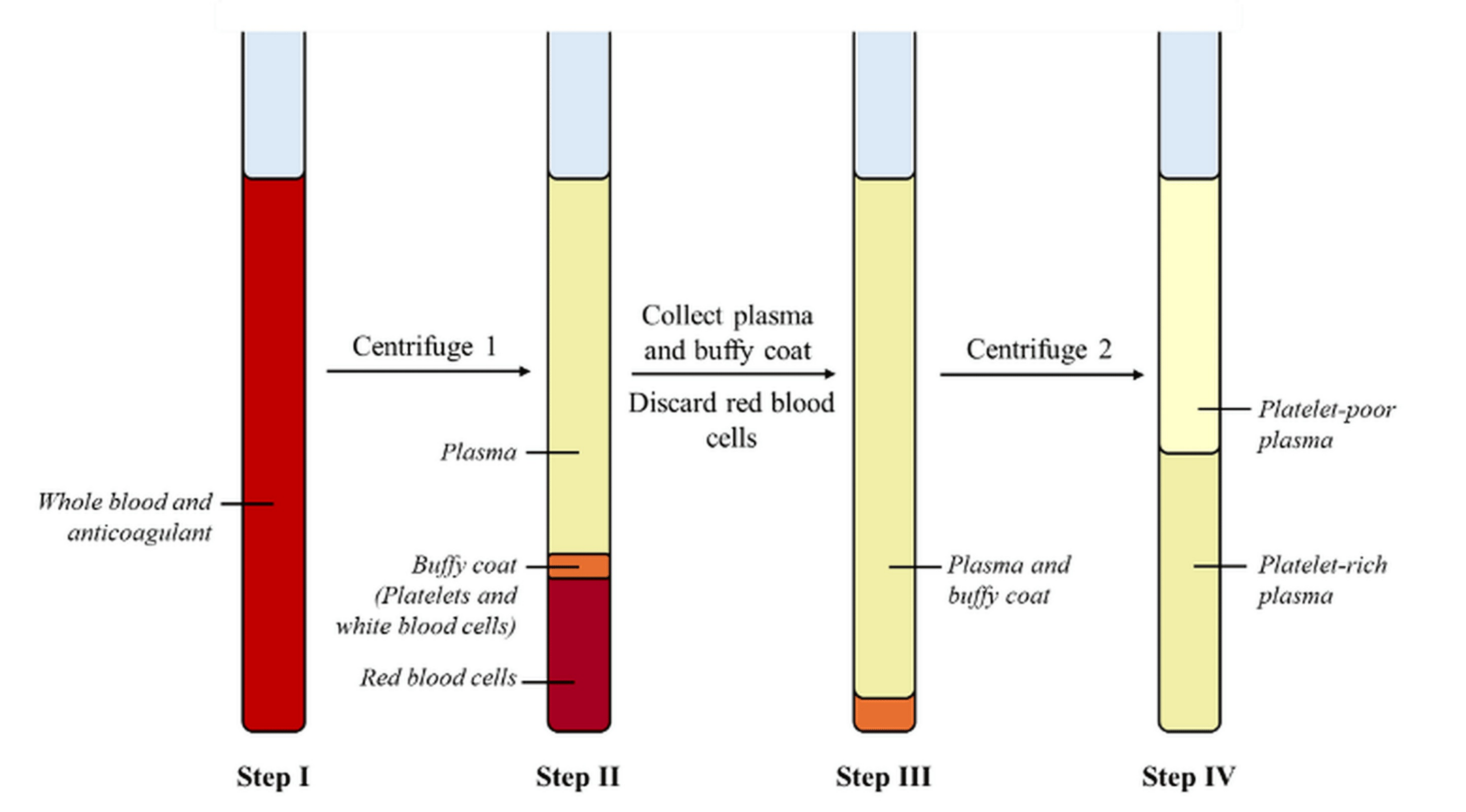
Schematic overview of platelet-rich plasma (PRP) preparation by the double-spin centrifugation method. Step I: Whole blood collected into an anticoagulant tube; Step II: First centrifugation separates components; Step III: Buffy coat and PRP layer transferred; Step IV: Second centrifugation concentrates PRP. Image credit: The image was created by the authors using Microsoft PowerPoint (Microsoft Corp., Redmond, WA, USA).

Heterologous PRP was prepared using T-LAB (T-LAB Regenerative Medicine; Istanbul, Turkey) kits and the vertical centrifuge available in our laboratory. PRP preparation by the double-spin centrifugation method is shown in Figure 2. Based on the centrifuge parameters available, the estimated relative centrifugal force was approximately 150 × g during the first centrifugation and approximately 300 × g during the second centrifugation.

Donor rats

**Figure 3:**
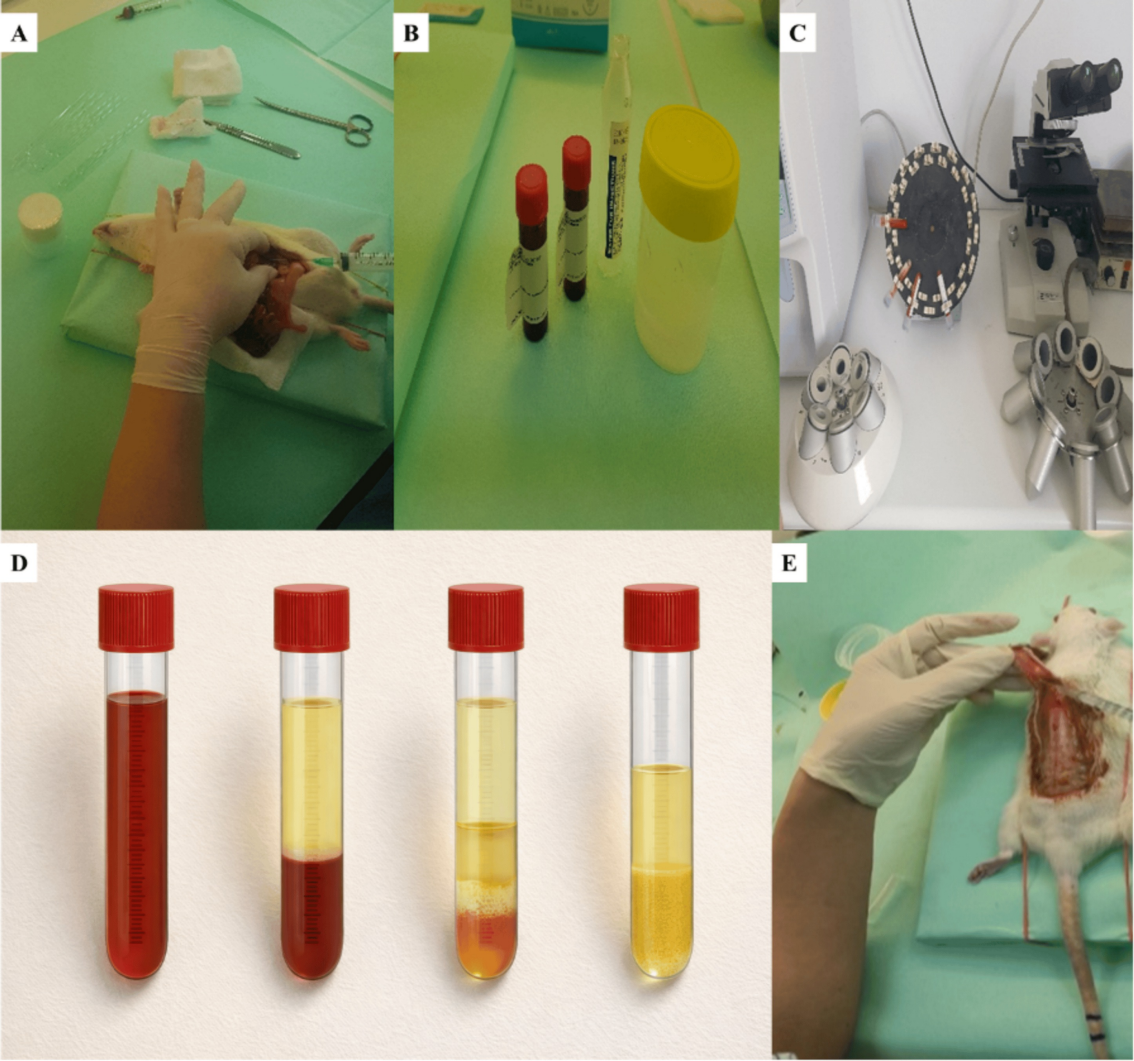
Experimental workflow for the random-pattern dorsal skin flap model and heterologous PRP preparation. A: Donor rat exsanguination setup for heterologous blood collection; B: Blood collection tubes and anticoagulant preparation for PRP production; C: Laboratory centrifugation equipment used for the double-spin protocol; D: Post-centrifugation separation of blood components; E: Intraoperative handling/processing associated with PRP preparation and/or flap procedure. PRP: platelet-rich plasma

Eight female donor rats were sedated and underwent complete exsanguination via laparotomy and catheterization of the vena cava (Figure 3A). Approximately 8.5 mL of blood per donor was collected into tubes preloaded with sodium citrate as an anticoagulant (Figure 3B) and processed immediately under indoor laboratory room conditions (approximately 25°C). The preparation followed a double-spin protocol (Figure 3C). The first centrifugation was performed at 2,400 rpm for 10 min. After this step, the upper plasma layer together with the buffy coat fraction was aspirated using a 21-gauge (21G) needle and transferred to a new tube without anticoagulant. A second centrifugation was then performed at 3,500 rpm for 15 min, after which the lower fraction was collected for administration. No exogenous activation step was performed before PRP administration (Figure 3D).

The final PRP product was not directly characterized by platelet count, leukocyte count, platelet enrichment factor, or growth factor quantification. Therefore, the intervention should be interpreted as heterologous PRP prepared under a fixed laboratory protocol rather than as a composition-defined biological formulation with a measured therapeutic threshold.

PRP administration

In Group A (the control group), no further intervention followed flap elevation. In Group B, heterologous PRP was administered immediately after flap elevation and prior to closure by local subcutaneous infiltration into the flap tissue (Figure 3E). A total volume of 1 mL PRP was administered per animal, divided into 10 injection sites of 0.1 mL each. Injection sites were uniformly distributed between the proximal and distal portions of the flap. PRP was delivered using a 21G needle. No topical placement was used.

Postoperative monitoring/euthanasia

Flaps were monitored on a daily basis. No rat died or experienced severe complications during the experiment. After eight days of monitoring, animals were euthanized using high-dose anesthesia (pentobarbital 200 mg/kg). The skin flaps were surgically excised, and the cranial aspect was marked for orientation. Flaps were photographed from both the superficial and deep surfaces, placed in 10% formalin, and submitted for histological analysis.

## Results

Histological analysis

**Figure 4:**
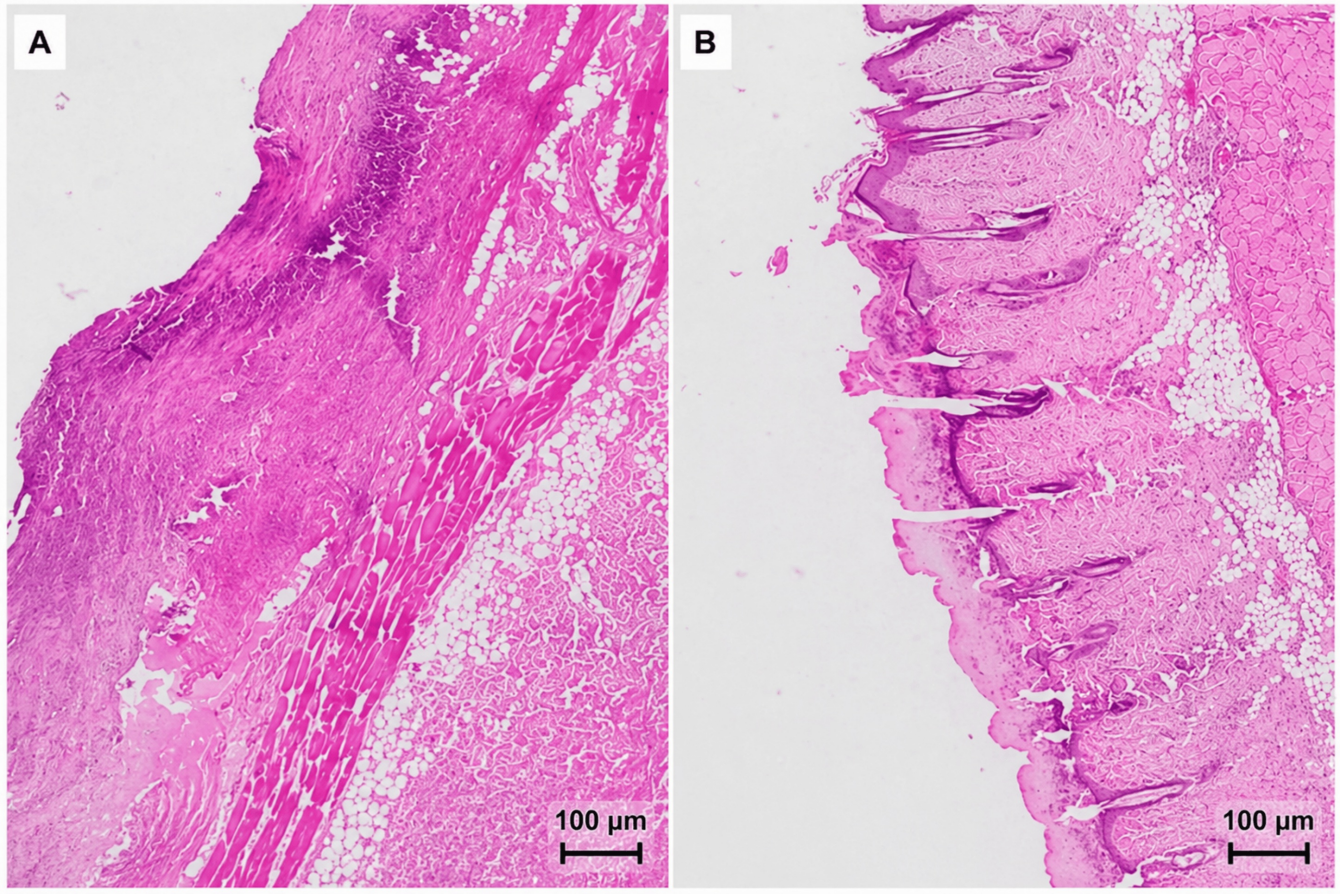
Representative H&E-stained sections from the distal portion of the dorsal skin flap for the two experimental groups. A: Control group, showing more extensive ischemic-necrotic injury with marked disruption of normal tissue architecture in the distal flap section. B: Heterologous PRP group, showing less extensive necrotic involvement and relatively greater preservation of tissue structure. H&E: hematoxylin and eosin; PRP: platelet-rich plasma

Tissue was harvested from the distal portion of each flap and processed using routine paraffin histology. Three-micrometer (3 µm) sections were cut from paraffin-embedded tissue, stained with hematoxylin and eosin (H&E), and examined under light microscopy. Necrosis percentage was assessed histologically on the basis of loss of normal tissue architecture and ischemic-necrotic changes involving the epidermal, dermal, and subcutaneous layers. The reported necrosis percentages represent observer-based estimates rather than automated digital area-fraction measurements. Histological assessment in the present study was focused on the extent of necrotic involvement. A formal semiquantitative scoring of additional features such as neoangiogenesis, collagen organization, microthrombi, or inflammatory cell subtype composition was not performed. Histological assessment was performed in a blinded manner (Figure 4).

Histological findings were subsequently compared between the two experimental groups: Group A (control group) and Group B (heterologous PRP). The percentage of necrosis affecting the evaluated layers was assessed and quantified by the histologist for each specimen (Table 1).

**Table 1:** Quantitative comparison of necrosis between groups.

Necrosis (%)	Group	Rat							
	A	1	2	3	4	5	6	7	8
		0.80	0.85	0.70	0.90	0.80	0.80	0.85	0.90
	B	1	2	3	4	5	6	7	8
		0.55	0.60	0.65	0.70	0.45	0.65	0.55	0.55

Normality assessment and between-group comparison

Before inferential testing, the distribution of necrosis percentage was assessed using the Shapiro-Wilk test and was consistent with normality (p = 0.130). Histological necrosis percentage differed significantly between groups (one-way ANOVA: F = 14.855, p < 0.001). Mean necrosis percentage was higher in the control group (0.82 ± 0.065) than in the heterologous PRP group (0.59 ± 0.079). The pairwise comparison between Group A and Group B was also significant (Tukey post hoc p < 0.001). Group-wise descriptive statistics and inferential results are summarized in Table 2.

**Table 2:** Summary of histological necrosis by group and between-group comparison. Data are shown as mean ± SD and range. Between-group comparison was performed using one-way ANOVA (F = 14.855, p < 0.001), followed by Tukey post hoc testing. The pairwise comparison between Group A and Group B was significant (p < 0.001). PRP: platelet-rich plasma; ANOVA: analysis of variance; SD: standard deviation

Group	n	Mean ± SD	Range	Between-group p-value
Control (Group A)	8	0.82 ± 0.065	0.70-0.90	ANOVA p < 0.001; Tukey A vs. B p < 0.001
Heterologous PRP (Group B)	8	0.59 ± 0.079	0.45-0.70	

Descriptive distribution of necrosis severity categories

**Figure 5:**
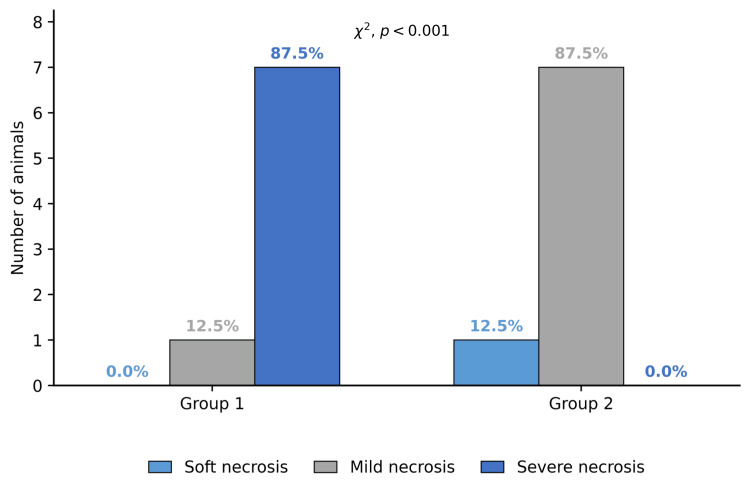
Descriptive distribution of necrosis percentage categories by group (n = 8 per group).

For descriptive illustration only, necrosis percentages were grouped into three percentage-based intervals (≤50%, >50% to ≤75%, and >75%) to visualize the distribution of injury severity across groups, corresponding descriptively to mild, moderate, and severe necrosis (Table 3 and Figure 5). These intervals were not based on a validated histological scoring system and were not used as the primary analytical outcome of the study. The principal statistical analysis was performed on the continuous necrosis percentage.

**Table 3:** Crosstabulation of necrosis category by group.

Necrosis	Group	
	A	B
Mild	0	1
Moderate	1	7
Severe	7	0

## Discussion

This study compared heterologous PRP with no additional intervention in a stringent random-pattern dorsal flap model and found significantly lower histological necrosis percentage in the PRP-treated group. Under the specific preparation and administration conditions used in this study, heterologous PRP was associated with improved distal flap viability compared with control. The categorized severity display was included only as a descriptive illustration of injury distribution, whereas the principal statistical interpretation is based on the continuous necrosis percentage outcome.

Our histological findings align with prior experimental evidence on PRP in ischemic flap models. Local PRP increased rabbit random flap survival to 74.4% (day 3) versus 65.8% control, reducing inflammation while enhancing vessel density [19]. Chai et al. similarly reported improved rat flap viability on days 5-7 [16]. Tian et al. demonstrated benefits in ultra-long (8 × 2 cm) dorsal flaps through elevated VEGF/PDGF and nitric oxide levels, mirroring our necrosis reduction [21]. PRP pretreatment protected mouse island flaps against ischemia-reperfusion injury, boosting survival and perfusion while suppressing reactive oxygen species (ROS), neutrophils, and cytokines (TNF-α, IL-1β, IL-6) via downregulated pASK-1/pNF-κB [22]. Sönmez et al. showed ischemia-potentiated PRP angiogenesis (↑VEGFR2 bioluminescence) with increased microvessel density [23]. Li et al.'s subcutaneous PRP (100 μL) in cranially based rat dorsal flaps raised survival to 61.2% versus 28-35.8% controls, paralleling our leukocyte-rich PRP (L-PRP) via ↑VEGF/PDGF, fewer inflammatory cells, and higher vascularity [24]. Intraoperative subflap PRP reduced necrosis while enhancing arterioles and neovascularization through VEGF/PDGF/TGF-β3 [25]. Finally, Su et al. confirmed that optimal PRP pretreatment deactivates JAK/STAT signaling, mitigating oxidative stress, inflammation, and apoptosis [26].

Limitations

Several limitations should be considered when interpreting the present findings. First, the sample size was modest (n = 16), which limits statistical power and generalizability. Second, evaluation was performed at a single postoperative time point (day 8), precluding dynamic assessment of flap perfusion and recovery over time. Third, the final PRP product was not directly characterized by platelet count, leukocyte count, platelet enrichment factor, or growth factor content. Accordingly, the intervention should be interpreted as protocol-defined rather than concentration-defined, and the study does not permit conclusions regarding the exact biological dose delivered or which PRP components were primarily responsible for the observed effect. Fourth, PRP preparation was performed under routine indoor laboratory conditions without prospective environmental temperature monitoring, which may have influenced platelet activation kinetics. Fifth, histopathological assessment was centered on necrosis percentage and did not include a formal qualitative or semiquantitative evaluation of neoangiogenesis, collagen fiber organization, microthrombi, or inflammatory infiltrate composition. Finally, the absence of direct platelet and leukocyte quantification limits the ability to define the biological dose of the administered PRP and restricts the reproducibility and mechanistic interpretation of the findings. Future studies should incorporate direct PRP characterization, standardized delivery metrics, broader histopathological endpoints, serial assessment, and larger cohorts.

## Conclusions

The present findings support an association between the applied heterologous PRP protocol and reduced distal flap necrosis percentage under the conditions tested. However, because the final PRP product was not directly characterized and additional qualitative and mechanistic histopathological parameters were not formally assessed, the findings should be interpreted as preliminary and protocol-specific rather than as evidence of a defined biological mechanism.
